# Ultrasonography in Canine Carpal Disorders

**DOI:** 10.1111/vru.70239

**Published:** 2026-09-17

**Authors:** Sven Klußmann, Andrea Meyer‐Lindenberg, Andreas Brühschwein

**Affiliations:** ^1^ Equine Clinic, Centre for Clinical Veterinary Medicine LMU Munich Oberschleißheim Germany; ^2^ Clinic of Small Animal Surgery and Reproduction, Centre for Clinical Veterinary Medicine LMU Munich Munich Germany

**Keywords:** joint effusion, ligament, musculoskeletal, soft tissue disorder, tendon

## Abstract

Ultrasonographic studies of canine carpal disorders are scarce and typically limited to case reports or evaluations of single structures. This prospective, single‐center study characterized ultrasonographic findings in canine carpal disorders confirmed by a composite reference standard and clinical follow‐up, and it assessed the diagnostic role of high‐frequency musculoskeletal ultrasound in relation to radiography and selectively performed cross‐sectional imaging. Forty‐eight carpi in 41 dogs were evaluated. This study represents the first ultrasound case series across the spectrum of canine carpal disorders. Musculotendinous disorders were most common (*n* = 30/48; 63%), followed by inflammatory arthropathy, neoplasia, and bone lesions (each 6/48; 13%). Ultrasound enabled compartmental localization of swelling and structural characterization of soft tissue abnormalities. Swelling distribution correlated with the underlying disorder in 46 of 48 (96%) carpi; subcutaneous thickening (*n* = 27/48; 56%) was frequent but nonspecific. Stenosing tenosynovitis of the abductor pollicis longus was the most common individual diagnosis (*n* = 16/48; 33%). Carpal effusion was identified sonographically and associated with dorsal displacement of the antebrachiocarpal fat pad. Ultrasonography did not allow reliable benign–malignant classification of mass lesions; therefore, definitive classification requires tissue sampling. In the subset undergoing cross‐sectional imaging, CT or MRI primarily refined lesion extent and osseous detail, particularly in bone lesions including fractures, but did not consistently alter the primary diagnosis established by combined radiography and ultrasonography. A combined radiographic and ultrasonographic approach is supported as a practical first‐line imaging strategy for canine carpal disease, with CT or MRI reserved for selected complex, equivocal, or predominantly osseous cases.

## 1 Introduction

The canine carpus is a complex region consisting of multiple bones, joints, and soft tissue structures [1, 2, 3]. Various conditions affecting these components can result in forelimb lameness [4], including fractures [5, 6, 7, 8, 9], luxations [10, 11, 12, 13, 14], ligamentous [15, 16, 17, 18, 19] and tendinous [20, 21, 22, 23] disorders, neoplasms [24, 25, 26, 27, 28], inflammatory arthropathies, and degenerative joint disease (DJD) [11, 29, 30]. Traumatic hyperextension, often caused by jumping or falling, frequently results in multiligamentous injuries involving the radiocarpal and carpometacarpal joints [31, 32, 33, 34, 35, 36]. Carpal disease is typically diagnosed using clinical and radiographic examinations, including stress views and arthrocentesis [4, 37, 38].

Radiography remains the first‐line imaging modality for assessing bone integrity, but it lacks the sensitivity required to detect soft tissue abnormalities, joint effusion, and early‐stage pathological changes [39]. Advanced techniques such as CT [40, 41], positron emission tomography‐computed tomography (PET‐CT) [42, 43], and MRI [25, 41, 44, 45, 46] allow more detailed evaluation in selected cases. Arthroscopy of the antebrachiocarpal joint has also been described, although its use is limited by invasiveness and specific indications [47]. In contrast, ultrasound is widely accessible, noninvasive, and dynamic, and does not require general anesthesia. It allows real‐time assessment and comparison with the contralateral limb [22, 48].

According to the European Society of Musculoskeletal Radiology (ESSR), ultrasound is the first‐line imaging modality for evaluating tendons, nerves, and soft tissues of the wrist in human medicine, as other imaging techniques rarely offer additional diagnostic value [49]. In dogs, ultrasound can be used to visualize normal carpal anatomy [50, 51, 52, 53] and intraspecific anatomical variations of the extensor tendons [54], as well as to diagnose individual pathologies such as stenosing tenosynovitis of the abductor pollicis longus muscle [55] or tendinopathy of the flexor carpi ulnaris muscle [21, 22]. To date, no comprehensive ultrasonographic study has addressed the spectrum of canine carpal disorders and the frequently associated nonspecific soft tissue swelling [4, 12, 13, 32, 35, 55, 56, 57].

The aims of this study were threefold: (1) to characterize ultrasonographic findings in canine carpal disorders confirmed by a composite reference standard and clinical follow‐up, (2) to analyze associated patterns of soft tissue swelling, and (3) to assess the clinical diagnostic role of ultrasound in relation to radiography and, where available, cross‐sectional imaging.

## 2 Materials and Methods

### 2.1 Selection and Description of Subjects

A prospective, single‐center case series of dogs with suspected carpal disease was conducted between 2018 and 2022 at the Clinic for Small Animal Surgery and Reproduction, Ludwig‐Maximilians‐University of Munich, Germany.

Each owner provided written consent for participation in the study as part of the treatment agreement. All examinations were performed according to clinical indications and standard protocols. No additional procedures were undertaken for study purposes. The study complied with European (Directive 2010/63/EU) and German (TierSchG, TierSchVersV) animal welfare regulations. As no legal or ethical violations occurred, approval from the Ethics and Animal Experimentation Committee was not required.

To be included, all dogs had to undergo general, neurologic, and orthopedic examinations and show evidence of carpal disease only. To ensure accurate localization of pathology to the carpus, concurrent disorders of the shoulder, elbow, or distal limb structures such as the metacarpus or digits were excluded by comprehensive orthopedic and neurologic examination, including range‐of‐motion testing, palpation for joint effusion, swelling, or pain, and radiographic assessment of the ipsilateral elbow, metacarpal region, and digits when indicated. All clinical examinations were performed by diplomates of the European College of Veterinary Surgeons (ECVS). Radiographs in at least two orthogonal projections and musculoskeletal ultrasonography of the affected carpus were mandatory. A definitive diagnosis of carpal disease, which served as the composite reference standard for final case classification, was established by integrating arthrocentesis results, microbiologic, cytologic, or histopathologic analyses, CT or MRI findings, surgical findings, and the clinical course over a minimum follow‐up period of 1 year.

The following sections describe the procedures for clinical assessment, imaging, diagnostic confirmation, and outcome evaluation.

### 2.2 Data Collection and Analysis

Data from signalment, clinical, radiographic, ultrasonographic, and cross‐sectional imaging (CT and MRI) examinations were collected and analyzed descriptively. Additional diagnostic data were collected, including the results of arthrocentesis, microbiologic, cytologic, and histopathologic analyses; surgical findings; treatment records; and long‐term clinical follow‐up (>1 year).

#### 2.2.1 Clinical Assessment

Clinical signs recorded included lameness, soft tissue swelling, arthralgia, pain on palpation, and joint effusion. Arthralgia was defined as pain elicited during passive or active motion of the carpal joint in the absence of visible swelling or palpable effusion.

Joint effusion was considered present when fluctuant distension of the carpal recess was palpable on physical examination.

#### 2.2.2 Radiographic Examination

Radiographs were obtained using a direct digital radiography system (Siemens Axiom Luminos dRF; Siemens Healthcare GmbH, Erlangen, Germany). Standard mediolateral and dorsopalmar views of the affected distal forelimb were obtained in all dogs. Additional stress radiographs were acquired when carpal instability was suspected on clinical examination (e.g., following traumatic hyperextension and/or in cases of suspected avulsion injury) and/or when standard radiographs were equivocal for carpal alignment.

Radiographs were assessed using a predefined structured assessment form, as outlined in Supporting Information S2. A radiographic diagnosis was recorded for each case. For standardized documentation, the carpus was divided into eight anatomic regions extending from the distal third of the antebrachium to the proximal third of the metacarpus. Findings were evaluated subjectively and graded using a predefined 4‐point scale (0 = *normal*; 1 = *mild*; 2 = *moderate*; 3 = *severe*).

#### 2.2.3 Ultrasonographic Examination

Ultrasonographic examinations were performed by two experienced investigators (S.K. and A.B.) using a GE LOGIQ E9 system (GE Healthcare, Solingen, Germany). A high‐frequency linear “Hockey Stick” transducer (L8–L18i‐D, 15–18 MHz) was used without a standoff pad. Depending on clinical requirements, dogs were examined awake, sedated, or under general anesthesia. Patients were positioned in lateral recumbency with the affected carpus uppermost.

The carpus was clipped, cleaned, degreased with alcohol, and coated with coupling gel. Ultrasonographic evaluation followed a stepwise, anatomy‐driven approach: (1) soft tissues, including tendons, muscles, and ligaments, were examined along their longitudinal course, and (2) osseous structures, including bone surfaces, joint spaces, and synovial recesses, were assessed. Both longitudinal and transverse scan planes were aligned either parallel or perpendicular to the fiber orientation or target structure. Image orientation was standardized (transducer surface at the top; cranial/medial to the left; caudal/lateral to the right), and ultrasound settings (gain, depth, and focus) were optimized for each structure. Each carpus was examined in neutral, extended, flexed, and dynamic positions. In dogs with unilateral disease, the contralateral limb was not routinely scanned because of owner constraints or limited patient cooperation.

For standardized documentation, each carpus was divided into 16 anatomic regions extending from the distal third of the antebrachium to the proximal third of the metacarpus and encompassing the cranial/dorsal, medial, lateral, and caudal/palmar aspects. Ultrasonographic findings were recorded using predefined structured assessment forms, as outlined in Supporting Information S3 and S4. These forms covered soft‐tissue, musculotendinous, ligamentous, osseous‐surface, articular, synovial, and mass‐associated abnormalities. Findings were graded subjectively using a predefined 4‐point severity scale (0 = *normal*; 1 = *mild*; 2 = *moderate*; 3 = *severe*). Because no validated quantitative criteria currently exist for the canine carpus, no side‐to‐side percentage differences or fixed quantitative thresholds, such as width or cross‐sectional area, were applied. An ultrasonographic diagnosis was recorded for each case.

#### 2.2.4 Final Confirmation of the Specific Carpal Disorder, Treatment, and Clinical Course

The final diagnosis for each carpus was established using a composite reference standard based on the integration of clinical, imaging, and diagnostic findings, including results of arthrocentesis, cytologic, microbiologic, and histopathologic analyses, as well as CT, MRI, surgical findings, and clinical follow‐up over a minimum period of 1 year. In 11 carpi, further tissue diagnostics or cross‐sectional imaging were not performed because of initial owner refusal. These cases were nonetheless included because the clinical presentation, radiographic and ultrasonographic findings, treatment response, and 1‐year follow‐up findings were considered sufficiently consistent to support final case classification within the composite reference standard.

Advanced imaging was pursued when clinical examination in combination with radiography and ultrasonography did not allow sufficient lesion localization or characterization to guide case management. The choice between CT and MRI was based on the working diagnosis after integration of the clinical findings and differential diagnoses (e.g., suspected occult or complex osseous injury vs. suspected predominantly soft tissue involvement). All scans were performed under general anesthesia with the dog positioned in a head‐up prone position. The region of interest extended from the distal third of the antebrachium to the middle third of the metacarpus.

CT examinations were performed using a 64‐slice helical CT scanner (SOMATOM Definition AS, CT070/13/S; Siemens Healthcare GmbH, Erlangen, Germany). Acquisition parameters included 140 mAs, 120 kV, a 512 × 512 image matrix, a reconstructed slice thickness of 0.6 mm, a reconstruction interval of 0.6 mm, a rotation time of 0.5 s, and reconstruction kernels B30s and B70s. A topogram was used to define the region of interest. Intravenous contrast enhancement was achieved using a nonionic, monomeric, triiodinated, water‐soluble contrast agent (iohexol; Accupaque 300; GE Healthcare Buchler GmbH & Co. KG, Braunschweig, Germany) administered at a dose of 2 mL/kg body weight via the cephalic vein using a fully automated contrast injector (MEDRAD Stellant; Bayer Vital GmbH, Bayer Healthcare Radiology, Leverkusen, Germany). A 5–20 mL bolus of 0.9% NaCl was administered before and after contrast injection to flush the catheter system, depending on body weight and catheter size. Injection flow rates ranged from 1.5 to 3.5 mL/s.

MRI examinations were performed using a 1.5‐T MRI system (MAGNETOM Symphony; Siemens, Erlangen, Germany) with the same region of interest. Imaging sequences included T2‐weighted turbo spin echo (T2W), T1‐weighted spin echo (T1W) before and after intravenous contrast administration, and T2‐weighted short tau inversion recovery (STIR). Contrast enhancement was achieved with gadoteric acid (Dotavision; b.e. imaging GmbH, Baden‐Baden, Germany) administered manually as a 0.3‐mL/kg body weight IV bolus followed by a 0.9% NaCl flush.

All DICOM datasets were viewed and analyzed using Horos open‐source medical imaging software (version 3.3.6; www.horosproject.org; 2022 Horos Project).

Treatment strategies were documented but were not standardized, reflecting the observational nature of the study. Clinical outcomes were assessed at 1 year on the basis of follow‐up examination and owner feedback and were classified into four categories:

- No lameness/symptom‐free
- Residual lameness with improvement
- Persistent lameness/symptomatic without improvement
- Euthanasia due to progression of carpal disease or another reason

### 2.3 Statistics

All data were recorded and organized in Microsoft Excel (Microsoft 365 Excel, 2024; Microsoft Corporation, Redmond, WA, USA). Statistical analyses were performed in consultation with a state‐certified senior medical statistician using the R statistical software environment (version 4.3; R Foundation for Statistical Computing, Vienna, Austria).

For overview and comparison, each carpus was classified into one of four mutually exclusive diagnostic categories based on the final confirmed diagnosis. The bone lesion category comprised cases in which the primary diagnosis was an osseous disorder, irrespective of any concurrent articular reaction. Because DJD frequently coexisted with other primary diagnoses and may be accompanied by variable articular inflammation or effusion, it was recorded as a concurrent degenerative finding (e.g., periarticular osteophytosis and subchondral bone remodeling/sclerosis) rather than as a separate primary category.

- Inflammatory arthropathy (suppurative or nonsuppurative)
- Bone lesion
- Neoplasia
- Musculotendinous disorder

Descriptive statistics, including means and standard deviations, were calculated. Inferential statistics were not used because of the observational study design and limited sample size.

## 3 Results

The following sections summarize the key findings. Detailed results for individual imaging findings on ultrasonography and radiography are provided in Supporting Information S1–S8. Percentages are based on the total cohort (*n* = 48) for carpus‐level results, on the number of dogs (*n* = 41) for demographic and outcome data, and on the stated denominators for modality‐specific subsets.

### 3.1 Study Population

A total of 41 dogs, all classified as companion animals, with 48 affected carpi met all inclusion criteria. Seven of 41 (17%) dogs were affected bilaterally, 20 of 41 (49%) had a unilateral lesion on the left side, and 14 of 41 (34%) had a unilateral lesion on the right side. The mean age at ultrasonographic examination was 7.1 ± 3.2 years (range: 1–12.3), and the mean body weight was 27.4 ± 11.8 kg (range: 5.1–50.1).

The study population included 14 of 41 (34%) intact males, 14 of 41 (34%) neutered males, 6 of 41 (15%) intact females, and 7 of 41 (17%) spayed females.

The most common breeds were crossbreeds (*n* = 12/41; 29%), Golden Retrievers (*n* = 3/41, 7%), and Labrador Retrievers (*n* = 3/41, 7%). Because of the limited breed representation, statistical analysis between breeds was not performed.

### 3.2 Clinical Signs, Treatment, and Outcome

The most frequently reported clinical signs were a combination of lameness and soft tissue swelling (*n* = 19/48; 40%), lameness alone (*n* = 17/48; 35%), and swelling alone (*n* = 9/48; 19%). Arthralgia was noted less frequently (*n* = 3/48; 6 %). In the majority of cases (*n* = 40/48; 83%), no underlying traumatic, infectious, degenerative, or neoplastic cause of the clinical signs was identified at presentation.

Orthopedic examination revealed soft tissue swelling in 37 of 48 (77%) carpi, lameness in 34 of 48 (71%), and pain on palpation in 32 of 48 (67%). Palpable joint effusion was observed in 7 of 48 (15%) carpi.

Most carpi (*n* = 34/48; 70%) were managed conservatively, including NSAID therapy, immobilization, and/or peritendinous triamcinolone acetonide injection. At 1‐year follow‐up, 22 of 48 (46%) carpi were asymptomatic, and 18 of 48 (38%) showed clinical improvement. Three of 41 (7%) dogs were euthanized within a year: one dog due to diagnosed neoplasia of the carpus, and two dogs due to non‐carpus‐related diseases—intoxication and urothelial carcinoma of the urinary bladder.

Treatment protocols and long‐term outcomes are summarized in Tables 1 and 2.

**TABLE 1:** Treatment information per category.

Treatment	Inflammatory arthropathy (*n* = 6)	Bone lesion (*n* = 6)	Neoplasia (*n* = 6)	Musculotendinous disorder (*n* = 30).
No treatment	0 (0%)	2 (33%)	1 (17%)	0 (0%)
NSAID, movement restriction, supporting and immobilizing bandage	6 (100%)	1 (17%)	1 (17%)	21 (70%)
Peritendinous injection of triamcinolone acetonide	0 (0%)	0 (0%)	0 (0%)	5 (17%)
Surgical treatment	0 (0%)	3 (50%)	1 (17%)	4 (13%)
Radiotherapy	0 (0%)	0 (0%)	2 (33%)	0 (0%)
Radiotherapy and surgical treatment	0 (0%)	0 (0%)	1 (17%)	0 (0%)

**TABLE 2:** The results of the clinical course for at least 1 year per category.

Result (clinical course)	Inflammatory arthropathy *n* = 6	Bone lesion *n* = 6	Neoplasia *n* = 6	Musculotendinous disorder *n* = 30
No lameness, symptom‐free	2 (33%)	1 (17%)	1 (17%)	18 (60%)
Residual lameness, improvement	4 (67%)	1 (17%)	2 (33%)	11 (37%)
Lameness; no improvement	0 (0%)	0 (0%)	2 (33%)	1 (3.3%)
Euthanasia	0 (0%)	4 (67%)	1 (17%)	0 (0%)

### 3.3 Diagnostic Categories and Confirmation of Final Diagnosis

**FIGURE 1:**
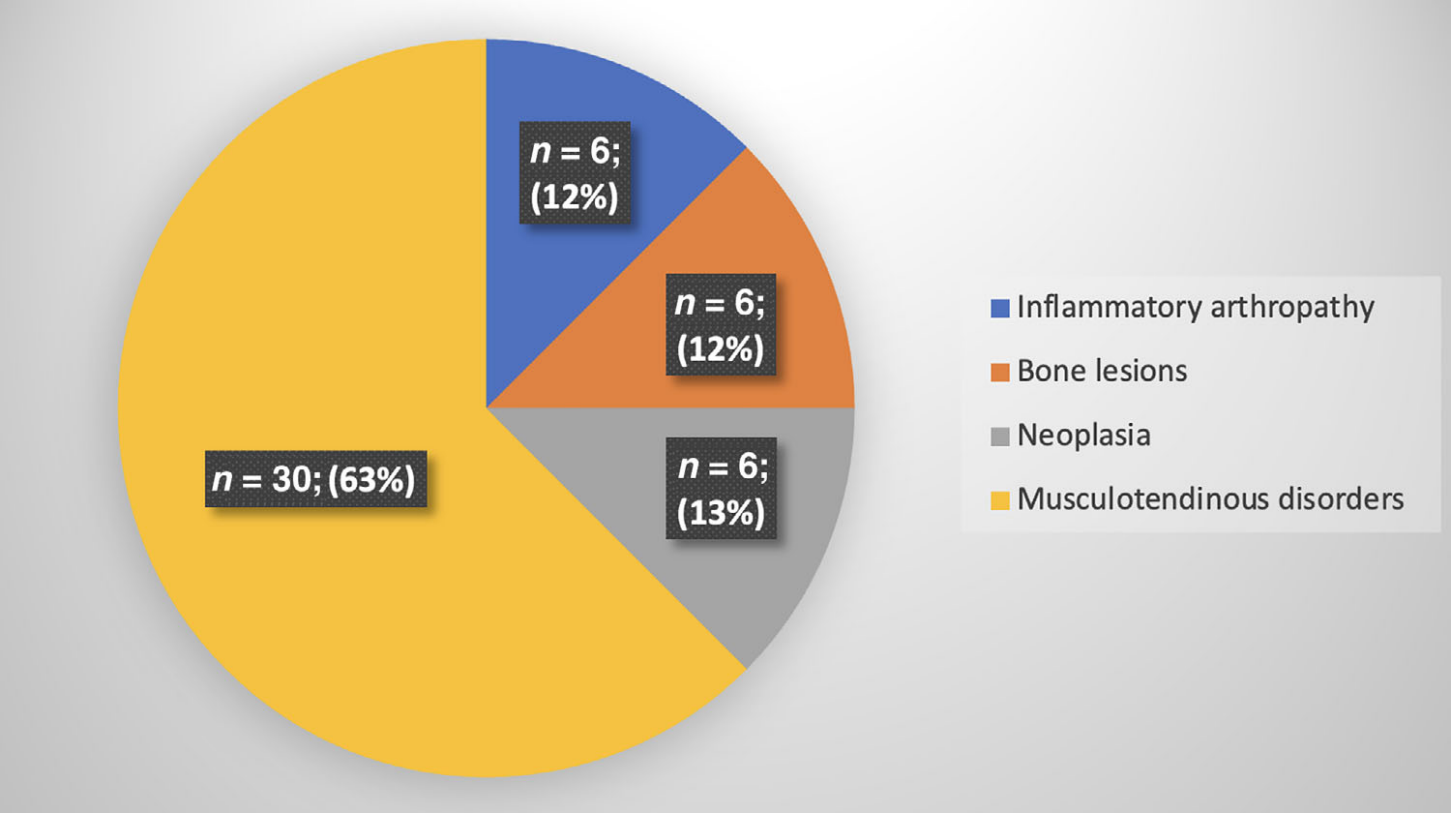
Etiology of carpal disease per category.

All 48 carpi were classified into one of four diagnostic categories based on the final confirmed diagnosis: musculotendinous disorders (*n* = 30/48; 63%), inflammatory arthropathy (*n* = 6/48; 13%), bone lesions (*n* = 6/48; 13%), and neoplasia (*n* = 6/48; 13%) (Figure 1).

US was performed on 28 of 48 (58%) carpi while the dogs were awake and on 20 of 48 (42%) carpi under general anesthesia. Twenty‐seven of 48 (56%) carpi were on the left side, and 21 of 48 (44%) were on the right side.

Final case classification was supported by one or more additional diagnostic methods in 37 of 48 (77%) carpi, most commonly cytology (*n* = 21/48; 43%), microbiology (*n* = 10/48; 21%), histopathology (*n* = 7/48; 15%), surgical exploration (*n* = 9/48; 19%), CT (*n* = 17/48; 35%), and MRI (*n* = 3/48; 6%). One case of 48 (2%) underwent both CT and MRI. Inflammatory arthropathy (*n* = 6/48; 13%) was confirmed by arthrocentesis, cytology, microbiology, histopathology, or combinations thereof.

In the remaining 11 of 48 (23%) carpi, all within the musculotendinous category, final classification was based on integrated clinical and imaging findings, treatment response, and 1‐year follow‐up within the predefined composite reference standard. The required combinations of methods for each final diagnosis are described in Table 3.

**TABLE 3:** Summary of final diagnoses, methods of confirmation, affected side, and diagnostic category.

Nr.	Cat.	Side	Final Diagnosis	ME	S	C/HE	CT	MRI
1	4	L	Chronic fibrosing granulating tendovaginitis and dystrophic calcifying tendinitis of the EDC	X	X	H	X	
2	4	L	Strain of the FCU, sprain of the AMLIV+V					
3	1	L	IMPA	X		C		
4	4	L	Stenosing tenosynovitis of the abductor pollicis longus muscle					X
5	3	R	Hidrocystoma	X	X	H		
6	4	R	Sterile purulent tenosynovitis of the ECR, DJD	X	X	H	X	
7	4	R	Sterile fibrinous tenosynovitis of the DDFM			C		
8	4	L	Stenosing tenosynovitis of the abductor pollicis longus muscle			C	X	
9	1	R	Pericarpal edema, hemarthrosis	X		C		
10	4	R	Musculotendinopathy of the FCU					
11	2	R	Fracture McV, DJD, enthesopathy AMLV, sec. joint effusion		X	C	X	
12	4	L	Enthesopathy of the ECR and AMLV				X	X
13	4	R	Stenosing tenosynovitis of the abductor pollicis longus muscle			C	X	
14	4	L	Stenosing tenosynovitis of the abductor pollicis longus muscle, metaplasia MCL				X	
15	4	R	Tendovaginitis of the SDFM, IMPA			C		X
16	4	L	Stenosing tenosynovitis of the abductor pollicis longus muscle, DJD					
17	4	R	Stenosing tenosynovitis of the abductor pollicis longus muscle, DJD					
18	4	R	Stenosing tenosynovitis of the abductor pollicis longus muscle					
19	4	L	Stenosing tenosynovitis of the abductor pollicis longus muscle					
20	4	R	Stenosing tenosynovitis of the abductor pollicis longus muscle					
21	3	R	Sarcoma		X	C		
22	1	L	Nonerosive immune‐mediated arthritis, SLE			C		
23	1	R	Nonerosive immune‐mediated arthritis, SLE			C		
24	1	R	Suppurative arthritis, stenosing tenosynovitis of the abductor pollicis longus muscle	X		C		
25	4	L	Musculotendinopathy of the FCU, enthesopathy of the AMLIV+V					
26	4	L	Musculotendinopathy of the ECR, DJD				X	
27	2	L	Complex fracture		X		X	
28	2	R	Complex fracture		X		X	
29	4	L	Unspecific joint effusion, edema, desmopathy of the AMLIV+V				X	
30	2	L	HO, urothelial carcinoma of the urinary bladder			C	X	
31	2	R	HO, urothelial carcinoma of the urinary bladder			C	X	
32	4	L	Musculotendinopathy of the FCU, desmopathy of the AMLV	X	X			
33	4	L	Stenosing tenosynovitis of the abductor pollicis longus muscle					
34	4	R	Stenosing tenosynovitis of the abductor pollicis longus muscle					
35	1	L	IMPA, DJD			C		
36	4	R	Stenosing tenosynovitis of the abductor pollicis longus muscle			C		
37	4	L	Stenosing tenosynovitis of the abductor pollicis longus muscle			C		
38	4	R	Stenosing tenosynovitis of the abductor pollicis longus muscle			C		
39	4	L	Stenosing tenosynovitis of the abductor pollicis longus muscle					
40	2	L	Exogenous bacterial osteomyelitis	X		H	X	
41	3	R	Sarcoma			C	X	
42	3	L	Sarcoma			C	X	
43	3	L	Fibrolipoma			C	X	
44	4	L	Fibrosing tenosynovitis of the SDFM			H		
45	4	L	Stenosing tenosynovitis of the abductor pollicis longus muscle, hygroma	X		H		
46	4	L	Sprain of the AMLIV+V	X	X			
47	3	L	Infiltrating lipoma			H		
48	4	R	Fibrosing tenosynovitis of the FCU			C		
			Total number:	10	9	28	17	3

Abbreviations: AMLIV, accessoriometacarpal ligament IV; AMLV, accessoriometacarpal ligament V; C/HE, cytological/histopathological examination; Cat., category (1: inflammatory arthropathy; 2: bone lesion; 3: neoplasia; 4: musculotendinous disorder); DDFM, deep digital flexor muscle; DJD, degenerative joint disease; ECR, extensor carpi radialis; EDC, extensor digitorum communis; FCU, flexor carpi ulnaris; HO, hypertrophic osteopathy; IMPA, immune‐mediated polyarthritis; Mc V, metacarpus V; MCL, medial collateral ligament; ME, microbiological examination; Nr., number; SDFM, superficial digital flexor muscle; SLE, systemic lupus erythematosus.

### 3.4 Inflammatory Arthropathy Category (*n* = 6/48; 13%)

Four of 48 (8%) cases were immune‐mediated, while 2 of 48 (4%) were nonimmune: one traumatic hemarthrosis and one bacterial arthritis with concurrent incidental stenosing tenosynovitis of the abductor pollicis longus muscle.

Radiographically, all carpi showed generalized soft tissue swelling. Mild signs of DJD were present in 4 of 48 (8%) cases. The diagnoses were classified as nonspecific soft tissue disorders (*n* = 4/48; 8%), stenosing tenosynovitis (*n* = 1/48; 2%), or unremarkable (*n* = 1/48; 2%).

**FIGURE 2:**
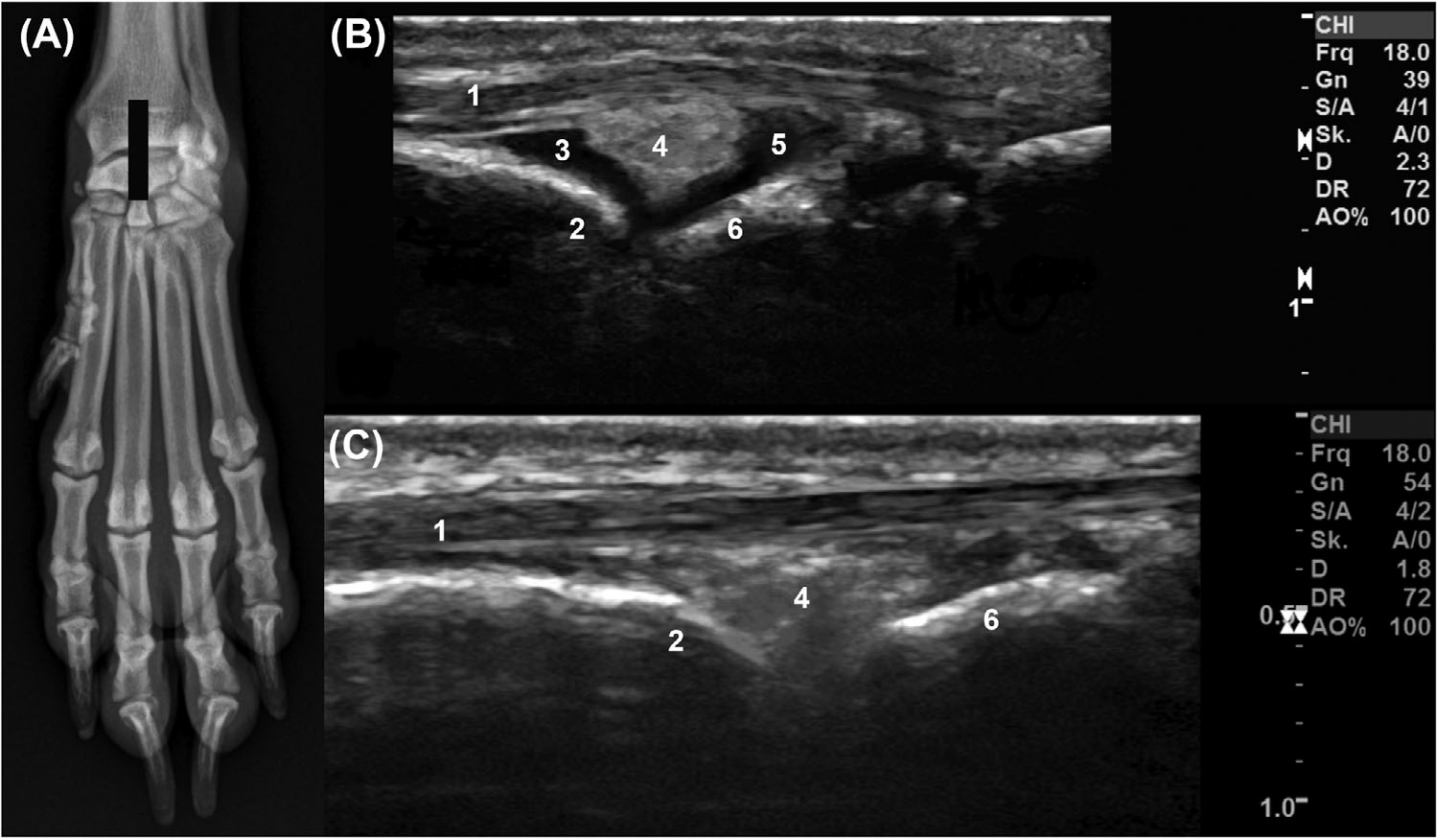
Anatomic reference and ultrasonographic appearance of antebrachiocarpal joint effusion. (A) Dorsopalmar radiograph of the carpus used as an anatomic reference. The broad vertical black line marks the approximate probe location and sagittal scanning plane at the level of the groove for the extensor carpi radialis tendon and the intermedioradial carpal bone. (B) Sagittal ultrasonographic image of the distended dorsal antebrachiocarpal recess showing an anechoic joint effusion and dorsal displacement of the dorsal antebrachiocarpal fat pad. The capsular outline may be difficult to delineate directly; the effusion is seen as an anechoic band deep to the fat pad and parallels the expected capsular contour. (C) Normal sagittal ultrasonographic image of the antebrachiocarpal joint for comparison, showing the dorsal antebrachiocarpal fat pad in its normal position and absence of visible joint effusion. (1) Extensor carpi radialis brevis muscle; (2) groove for the extensor carpi radialis tendon; (3) dorsoproximal antebrachiocarpal recess; (4) dorsal antebrachiocarpal fat pad; (5) dorsodistal antebrachiocarpal recess; and (6) intermedioradial carpal bone.

Ultrasonographically, all 6 of 48 (13%) joints showed homogeneous anechoic effusion in the antebrachiocarpal, mediocarpal, and carpometacarpal joints, with dorsal displacement of the antebrachiocarpal fat pad (Figure 2). Four carpi (8%) showed additional subcutaneous thickening and mild degenerative changes. One case was also diagnosed with concurrent stenosing tenosynovitis. In the longitudinal plane, joint effusion formed a homogeneous anechoic band deep to the antebrachiocarpal fat pad, paralleling a smooth capsular line (Figure 2). Beyond effusion‐related capsular distension, no overt capsular thickening or synovial proliferative changes were identified.

Cross‐sectional imaging was not performed.

### 3.5 Bone Lesion Category (*n* = 6/48; 13%)

This group included 3 of 48 (6%) carpi with fractures, 2 of 48 (4%) carpi with hypertrophic osteopathy secondary to urothelial carcinoma of the urinary bladder, and 1 of 48 (2%) carpi with bacterial osteomyelitis.

Radiography and ultrasonography detected fractures in 2 of 48 (4%) carpi; however, the extent and complexity of these fractures could not be fully assessed. In 1 of 48 (2%) carpi, the fracture was not detected by either modality, and ultrasound showed only secondary cortical surface irregularity with adjacent soft tissue abnormalities. All fracture cases showed anechoic effusion in the carpometacarpal joint; the two complex fractures also involved the antebrachiocarpal and the mediocarpal joints. Ultrasonographically, the fractures appeared as localized disruptions of the hyperechoic cortical bone surface. Accurate assessment of the fracture lines and fragment position was not possible. Adjacent soft tissue abnormalities, including hematoma or edema, were noted in all cases.

**FIGURE 3:**
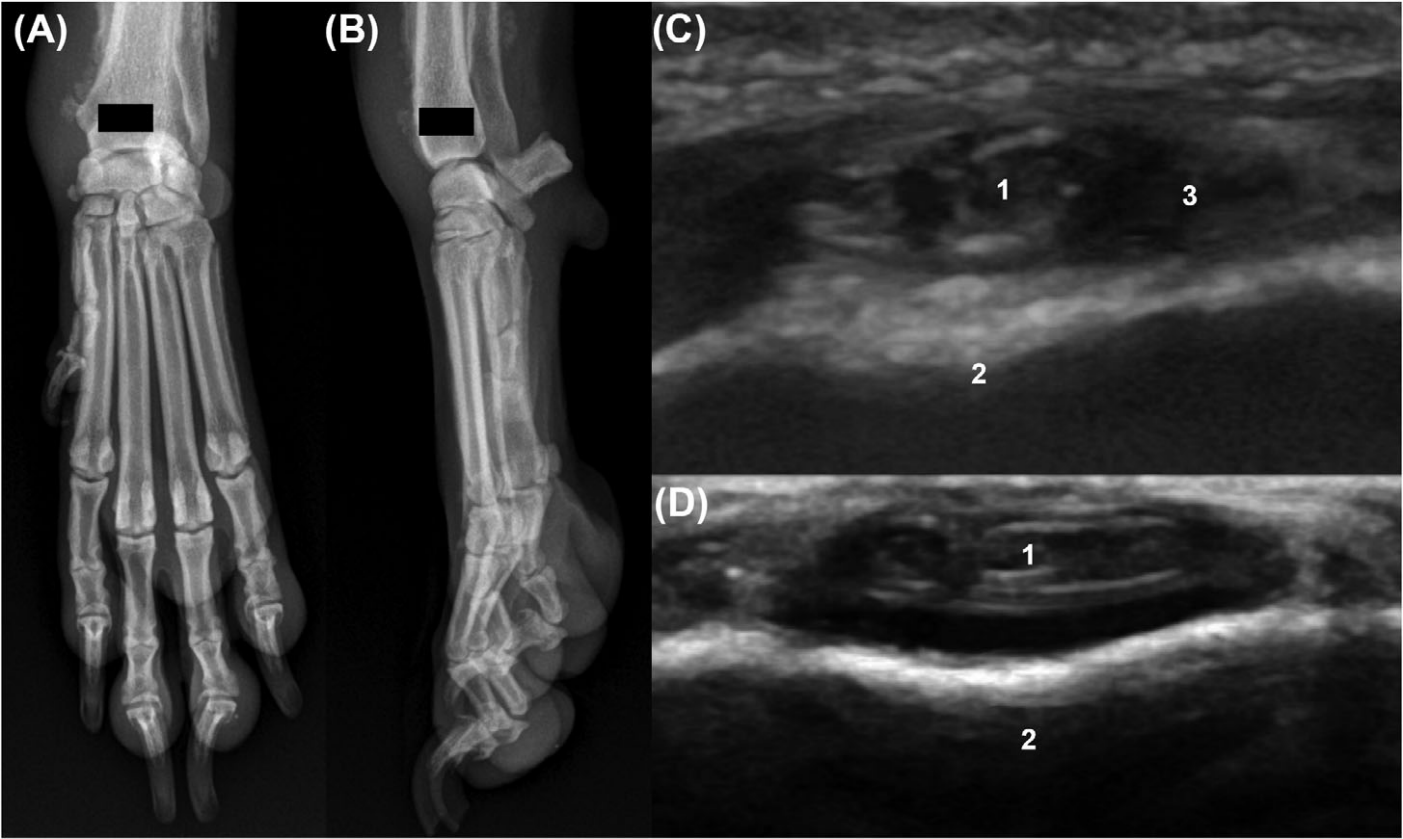
Anatomic reference and ultrasonographic appearance of hypertrophic osteopathy. (A, B) Dorsopalmar (A) and mediolateral (B) radiographs of the carpus used as anatomic references. The broad black line in each radiograph marks the approximate probe location and transverse scanning plane at the level of the groove for the extensor carpi radialis tendon. Smooth‐to‐lamellated periosteal new bone formation, consistent with periosteal “cuffing,” is present, with cortical thickening and regional soft‐tissue swelling. The periosteal proliferations extend to the level of the proximal groove for the extensor carpi radialis tendon. (C) Corresponding transverse ultrasound image at the proximal groove for the extensor carpi radialis tendon, demonstrating an irregular hyperechoic cortical surface with clean posterior acoustic shadowing. The cortical margin is rough and partially indistinct, and the groove for the extensor carpi radialis tendon is narrowed by periosteal apposition. The extensor carpi radialis tendon sheath is mildly distended, and the synovial lining is mildly thickened. (D) Normal transverse ultrasound image at the proximal groove for the extensor carpi radialis tendon for comparison, showing a smooth, sharply marginated hyperechoic cortical surface without periosteal apposition or narrowing of the groove. Anechoic antebrachiocarpal joint effusion is present. Numbers in C and D indicate (1) extensor carpi radialis tendon; (2) groove for the extensor carpi radialis tendon; and (3) mildly distended extensor carpi radialis tendon sheath with mildly thickened synovial lining.

Both cases of hypertrophic osteopathy and the case of osteomyelitis were identifiable on radiographs and ultrasound, presenting with periosteal roughening, cortical thickening, and adjacent soft tissue changes (Figure 3). In the longitudinal plane, cortical surface abnormalities were appreciable as irregularities or step‐offs of the echogenic cortical line, and periosteal proliferation formed uneven echogenic lamination along the bone surface.

CT was performed in all carpi in this category (*n* = 6/48; 13%), whereas MRI was not performed. CT diagnosed all fractures (*n* = 3/48; 6%) and delineated fracture configuration, fragment relationships, and articular involvement. In hypertrophic osteopathy (*n* = 2/48; 4%), CT characterized the distribution and surface morphology of periosteal new bone formation, including smooth‐to‐lamellated periosteal cuffing with preserved cortical integrity. In osteomyelitis (*n* = 1/48; 2%), CT showed cortical lysis, medullary involvement, and overall osseous extent. Compared with ultrasonography, CT identified regional soft tissue swelling but did not resolve soft tissue compartmentalization (subcutaneous, peritendinous, intra‐articular) or fluid characteristics, which were demonstrated on ultrasound.

### 3.6 Neoplasia Category (*n* = 6/48; 13%)

**FIGURE 4:**
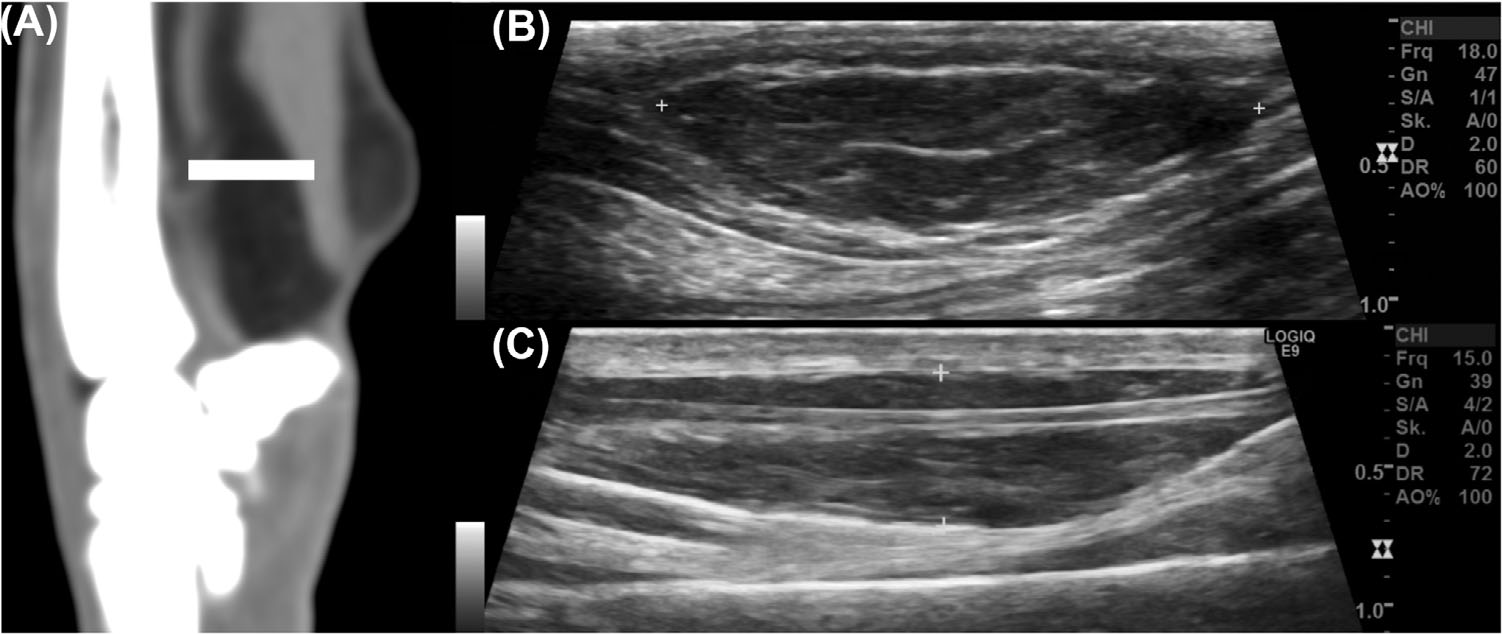
Anatomic reference and ultrasonographic appearance of a histopathologically confirmed infiltrative lipoma of the palmar carpal soft tissues. (A) Sagittal reconstructed CT image displayed in a soft‐tissue window shows a strand‐like, fat‐attenuating mass proximal to the accessory carpal bone; the broad horizontal white line through the mass marks the approximate probe location and transverse scanning plane corresponding to the ultrasound image in B. (B) Transverse ultrasound image of the palmar carpal soft tissues at the level indicated in A, showing a heterogeneous mass with mildly indistinct margins, marked with asterisks. (C) Corresponding sagittal ultrasound image showing the longitudinal extent of the heterogeneous, strand‐like palmar carpal mass, also marked with asterisks. Definite infiltrative growth is not evident on ultrasonography; the final diagnosis of infiltrative lipoma was established histopathologically.

This group included 3 of 48 (6%) sarcomas and 1 of 48 (2%) each of hidrocystoma, infiltrative lipoma (Figure 4), and fibrolipoma.

Radiographically, the most commonly affected areas were caudoproximal (area 6; *n* = 5/48; 10%) and palmarodistal (area 8; *n* = 4/48; 8%). The diagnoses included soft tissue mass lesions (*n* = 3/48; 6%), nonspecific soft tissue changes (*n* = 2/48; 4%), and one (2%) unremarkable case. Mild DJD was present in 1 of 48 (2%) carpi.

**FIGURE 5:**
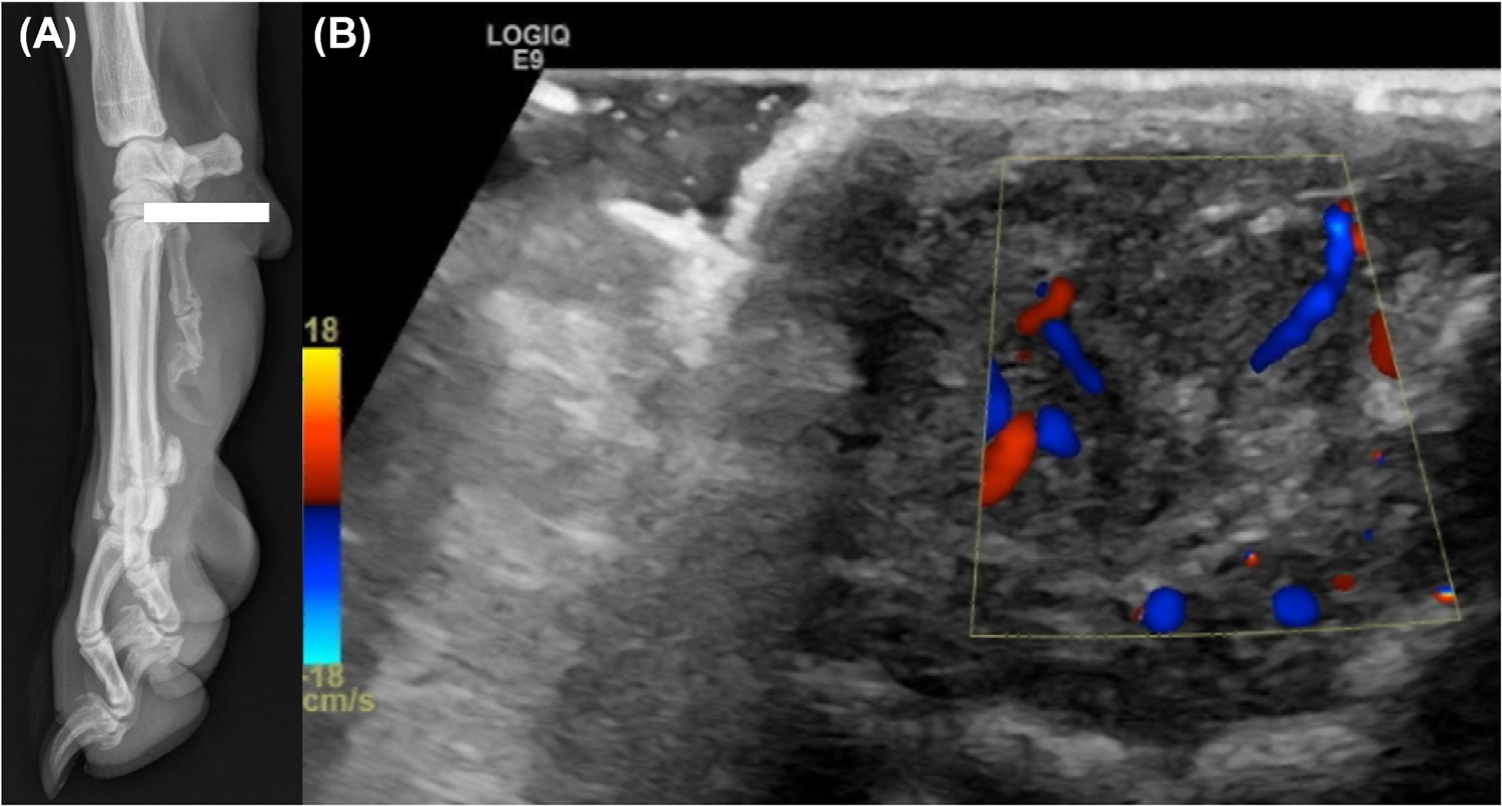
Anatomic reference and ultrasonographic appearance of a palmar carpal sarcoma. (A) Mediolateral radiograph of the carpus used as an anatomic reference, showing an elongated, convex palmar soft‐tissue swelling beginning in the carpal tunnel at the level of the accessory carpal bone and extending distally to the distal metacarpus. The broad horizontal white line marks the approximate probe location and transverse scanning plane corresponding to the ultrasound image in B. (B) Transverse ultrasound image at the level indicated in A, showing a poorly to moderately marginated, heterogeneous mass with mixed echogenicity and increased internal and peripheral vascularity. No internal acoustic shadowing is present within the mass; marginal edge shadowing is visible at the lesion interface. Ultrasound identified a mass lesion; sarcoma was confirmed cytologically.

On ultrasound, all masses showed heterogeneous echotexture and mixed echogenicity, lacked acoustic shadowing, and were poorly to moderately demarcated. Except for the hidrocystoma, all lesions exhibited increased vascularity (Figure 5). Half of the cases (*n* = 3/48; 6%) had concurrent subcutaneous thickening with heterogeneous echogenicity. The most frequently altered ultrasonographic regions were palmaromedial and palmarolateral (areas 14 and 16; *n* = 5/48; 10% each). The tissue compartment of origin could not be determined reliably in all cases. All neoplasms were correctly identified as mass lesions on ultrasound, and no concurrent musculotendinous abnormalities were present.

CT was performed in 3 of 48 (6%) carpi, whereas MRI was not performed. CT provided additional information on lesion size, internal architecture, attenuation‐ and enhancement‐based internal composition, and osseous involvement. Sarcomas appeared as heterogeneously enhancing soft tissue masses, whereas the lipomatous tumor showed fat‐attenuating components with variable soft tissue portions. No mass lesion was detected exclusively by CT.

### 3.7 Musculotendinous Disorders Category (*n* = 30/48; 63%)

This category included 30 of 48 (63%) carpi with various musculotendinous pathologies. In some cases, multiple structures were affected simultaneously (Table 3). Where contralateral images were available, they were used as a qualitative comparator only; no numeric thresholds were used.

#### 3.7.1 Radiographic Findings

Across all carpi, soft tissue swelling was observed in 28 of 48 (58%) carpi and was classified as diffuse in 22 of 48 (46%) cases. Radiographic diagnoses included stenosing tenosynovitis of the abductor pollicis longus muscle (*n* = 16; 33%), nonspecific soft tissue disorders (*n* = 12; 25%), mass lesion (*n* = 1; 2%), and unremarkable joint (*n* = 1; 2%). Mild DJD was present in 4 of 48 (8%) carpi.

#### 3.7.2 Ultrasonographic Findings

Overall, the primary ultrasonographic characteristics of musculotendinous lesions included heterogeneous volume increase and hypoechoic (*n* = 13/48; 27%) or mixed echogenicity (*n* = 17/48; 35%) of the affected anatomic structure. Subcutaneous tissue thickening with heterogeneous echogenicity was present in 15 of 48 (31%) carpi. Reliable differentiation between interstitial edema and subcutaneous fibrosis was not possible. In the longitudinal plane, affected tendons and ligaments showed thickening and loss of the normal parallel fibrillar pattern; tendon‐sheath distension, when present, extended longitudinally as an anechoic or hypoechoic column.

A specific musculotendinous diagnosis was established in 27 of 48 (56%) carpi based on ultrasound findings. Four of 48 (8%) carpi additionally showed mild homogeneous anechoic effusion in the antebrachiocarpal joint along with mild signs of DJD. Three of 48 (6%) carpi were classified as mass lesions, two of which showed increased vascularity of the affected structure.

Cross‐sectional imaging was obtained in a subset of musculotendinous disorders, including CT in 8 of 48 (17%) carpi and MRI in 3 of 48 (6%) carpi. In this subset, CT delineated mineralized or ossified components, enthesophytes, periosteal new bone at insertion sites, and cortical remodeling or irregularities, including APLM‐associated osseous changes such as cortical remodeling accentuating the medial radial sulcus and enthesophytes at the first metacarpal base. MRI outlined the extent of tenosynovitis. Fine soft tissue compartmentalization and nonmineralized abnormalities, including sheath thickening or distension, peritendinous or subcutaneous fluid, and intratendinous fiber derangement, were not resolved on CT and remained ultrasonographic findings. No CT‐only or MRI‐only primary musculotendinous diagnosis was recorded.

The most frequently affected structures are described in detail in the following subsections. Thickening was recorded using the predefined qualitative 4‐point scale; no side‐to‐side percentages or fixed quantitative cutoffs were applied.

#### 3.7.3 Abductor Pollicis Longus Muscle Changes (APLM, *n* = 16/48; 33%)

The APLM was the most frequently affected anatomic structure in this study, with stenosing tenosynovitis diagnosed in 16 of 48 (33%) carpi. Four of 41 (10%) dogs were affected bilaterally, and 8 of 41 (20%) dogs were affected unilaterally, with no side preference.

Radiographically, the predominant finding was a diffuse increase in soft tissue volume in the proximomedial area, observed in 12 of 48 (25%) carpi. Bony changes were present in 9 of 48 (19%) carpi, including focal or extensive osteophyte formation within or around the radial groove. In some cases, irregular, tubular proliferations extended beyond the styloid process. A deepened medial sulcus with radiopaque margins was visible in another 9 of 48 (19%) carpi. Two of 48 (4%) carpi showed enthesopathic changes at the base of the first metacarpal bone, accompanied by periosteal reactions on the proximomedial aspect.

**FIGURE 6:**
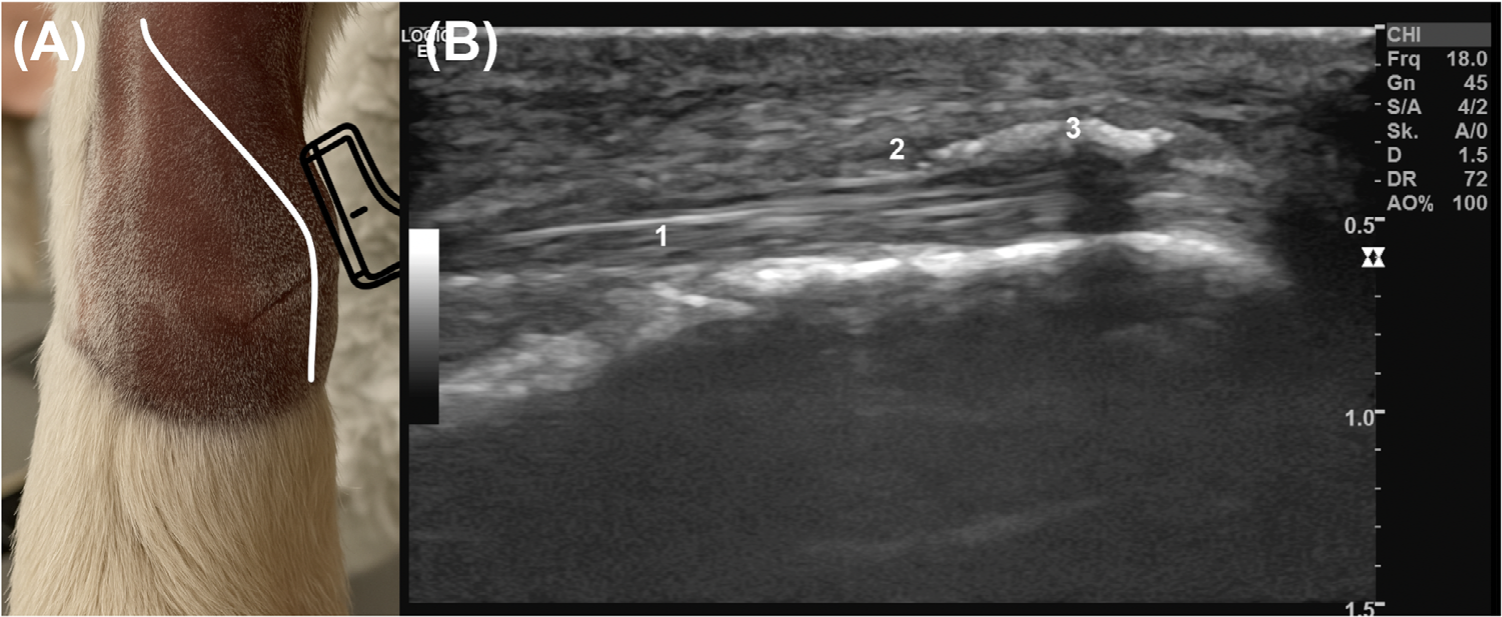
Anatomic reference and ultrasonographic appearance of stenosing tenosynovitis of the abductor pollicis longus tendon. (A) Photograph of the dorsal aspect of the canine carpus used as an anatomic reference. The broad white line indicates the course of the abductor pollicis longus tendon and the sagittal scanning direction; the transducer indicates the approximate probe position corresponding to the ultrasound image in B. (B) Sagittal ultrasound image of the abductor pollicis longus tendon showing tenosynovitis with an irregularly thickened tendon sheath of mixed echogenicity. Hyperechoic foci within the tendon sheath produce focal acoustic shadowing. Numbers in B indicate (1) abductor pollicis longus tendon; (2) thickened tendon sheath; and (3) hyperechoic foci with posterior acoustic shadowing.

Ultrasonographically, areas 1 and 5 were the most frequently altered regions and consistently showed increased soft tissue volume. All APLM lesions exhibited heterogeneous echotexture and hypoechoic to mixed echogenicity. Fifteen of 48 (31%) carpi showed thickening of the tendon sheath with irregular contour and variable echogenicity. Anechoic fluid within the tendon sheath was noted in 7 of 48 (16%) cases. Six of 48 (13%) carpi showed hyperechoic foci with acoustic shadowing (Figure 6), consistent with mineralization, while others contained nonshadowing hypoechoic inclusions. Enthesopathic changes were present in 10 of 48 (21%) carpi. In the longitudinal plane at the APLM insertion, enthesopathic changes appeared as irregularities and small step‐offs of the echogenic cortical line with focal echogenic excrescences; mineralized foci produced clean posterior acoustic shadowing, and the tendon insertion showed focal thickening with loss of the sharply marginated fibrillar termination.

Focal anechoic fluid accumulations adjacent to the tendon sheath were observed less frequently (*n* = 4/48; 8%).

#### 3.7.4 Accessoriometacarpal Ligament IV (AMLIV, *n* = 7/48; 15%) and Accessoriometacarpal Ligament V Changes (AMLV, *n* = 4/48; 8%)

Lesions of the accessoriometacarpal ligaments were the second most frequently affected structures within the musculotendinous group, involving AMLIV in 7 of 48 (15%) carpi and AMLV in 4 of 48 (8%) carpi. Because of their anatomic proximity and frequent co‐involvement, both are described together.

Radiographically, soft tissue swelling was detected in areas corresponding to the anatomic course of AMLIV and AMLV, particularly in the palmarodistal and lateral zones (e.g., areas 12 and 16). One carpus showed rupture of AMLV with concurrent desmopathy of AMLIV. In the remaining cases, desmopathy with or without enthesopathy was identified, typically associated with periosteal irregularities near the ligament insertions.

**FIGURE 7:**
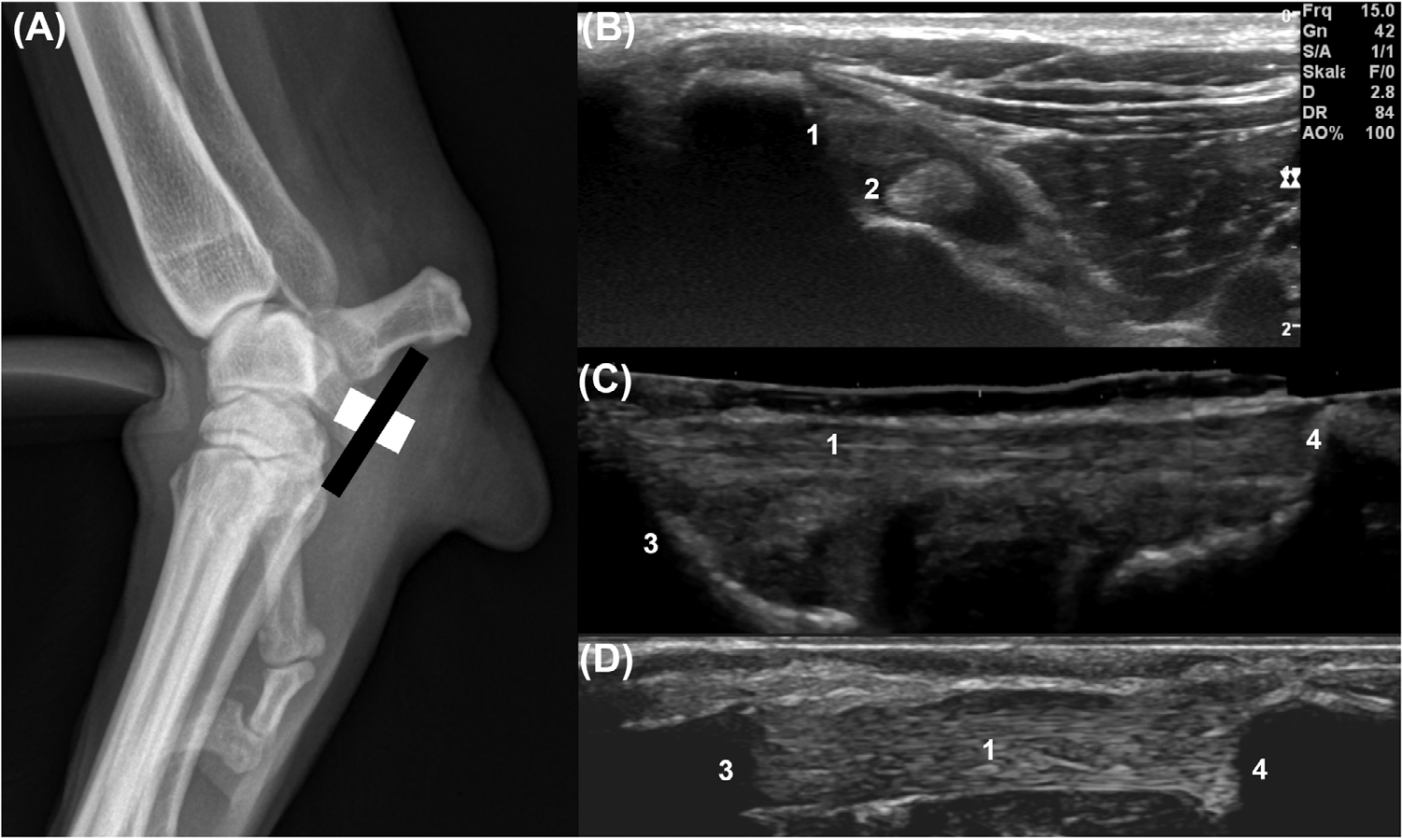
Anatomic reference and ultrasonographic appearance of trauma‐associated desmopathy of the accessoriometacarpal ligaments IV and V. (A) Mediolateral stress radiograph of the carpus used as an anatomic reference. The broad white line marks the approximate probe location and transverse scanning plane through the central portion of the accessoriometacarpal ligaments IV and V, corresponding to B. The broad black line marks the approximate probe location and sagittal scanning plane along the course of the accessoriometacarpal ligaments from the accessory carpal bone to the metacarpal bones, corresponding to C and D. (B) Transverse ultrasound image at the level indicated in A, showing a diffusely hypoechoic accessoriometacarpal ligament V, consistent with desmopathy, and a small adjacent anechoic periligamentous fluid accumulation. (C) Sagittal ultrasound image of the affected accessoriometacarpal ligament V, showing loss of the normal fibrillar echotexture. (D) Normal sagittal ultrasound image of the accessoriometacarpal ligament V for comparison. Numbers in B–D indicate (1) accessoriometacarpal ligament V; (2) accessoriometacarpal ligament IV; (3) accessory carpal bone; and (4) metacarpal V.

Ultrasonographically, the ligaments appeared thickened, with heterogeneous volume increase and hypoechoic to mixed echogenicity (Figure 7). Insertional irregularities were noted, and five carpi showed enthesopathic changes.

#### 3.7.5 Flexor Carpi Ulnaris Muscle Changes (FCUM, *n* = 5/48; 10%)

Musculotendinopathy was diagnosed in 5 of 48 (10%) carpi.

##### 3.7.5.1 Radiographic Findings

Soft tissue swelling was detected in areas corresponding to the anatomic location of the FCUM, most commonly in area 2 (proximolateral) and area 5 (cranioproximal). Two of 48 (4%) carpi showed mild enthesopathy, indicated by periosteal reactions at the insertion sites.

##### 3.7.5.2 Ultrasonographic Findings

**FIGURE 8:**
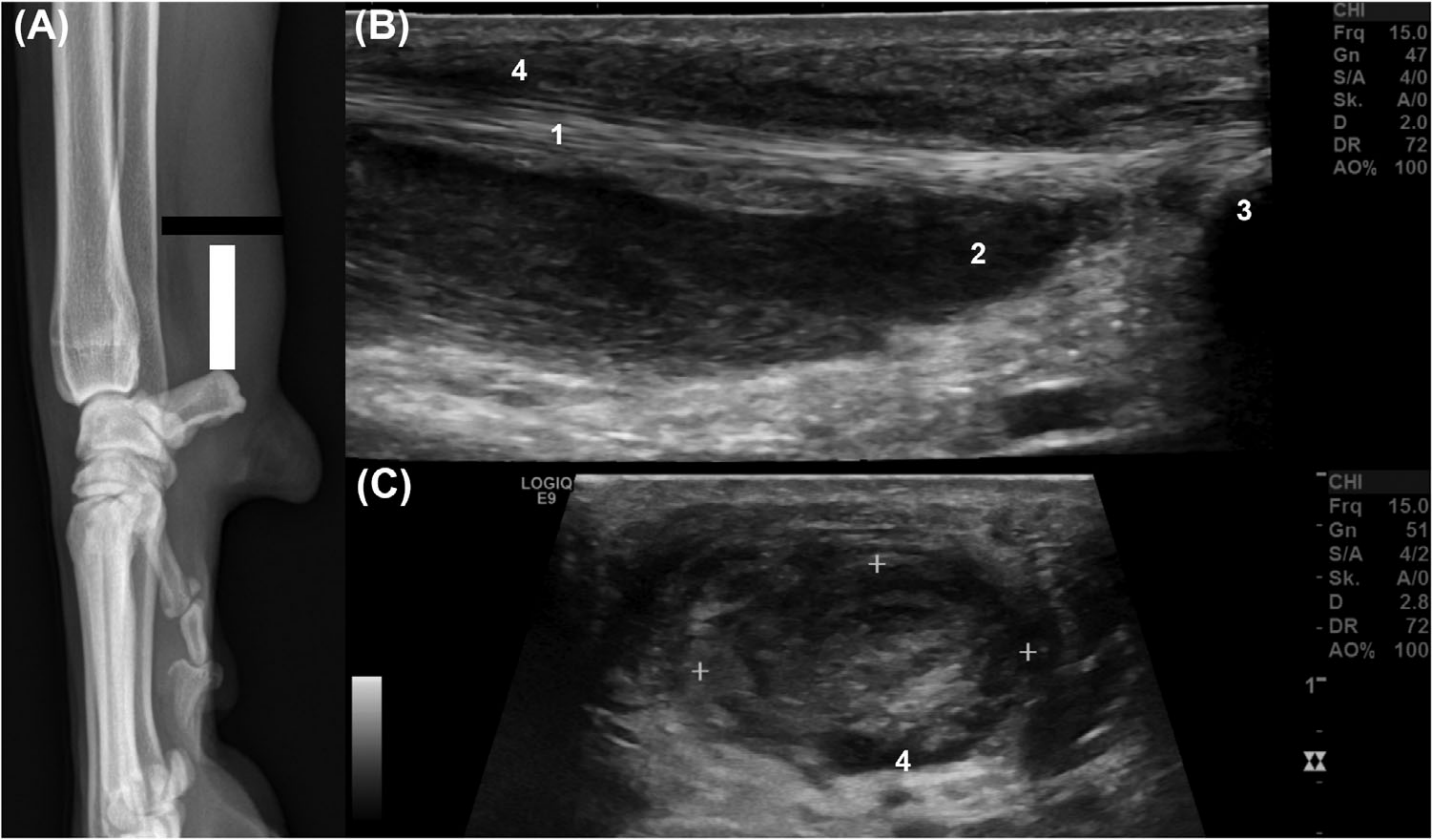
Anatomic reference and ultrasonographic appearance of severe musculotendinopathy of the flexor carpi ulnaris muscle. (A) Mediolateral radiograph of the carpus used as an anatomic reference. The broad white line marks the approximate probe location and sagittal scanning plane corresponding to the ultrasound image in B. The broad black line marks the approximate probe location and transverse scanning plane corresponding to the ultrasound image in C. A convex palmar soft‐tissue swelling is present proximal to the accessory carpal bone, with preservation of the soft‐tissue outline. (B) Sagittal ultrasound image of severe musculotendinopathy of the flexor carpi ulnaris muscle. The humeral head tendon is no longer clearly distinguishable and shows marked heterogeneous enlargement with loss of the normal fibrillar echotexture. The ulnar head tendon remains distinctly distinguishable but shows mild loss of the normal fibrillar echotexture, with an anechoic to hypoechoic region at its insertion on the accessory carpal bone. Associated peritendinous anechoic fluid and adjacent hypoechoic soft‐tissue thickening are present. (C) Transverse ultrasound image of the affected flexor carpi ulnaris muscle near the musculotendinous junction, marked with asterisks. The normal configuration of the combined tendon as a narrow, rounded structure is no longer distinguishable. Peritendinous anechoic fluid and adjacent hypoechoic soft‐tissue thickening are present. Numbers in B and C indicate (1) ulnar head tendon of the flexor carpi ulnaris muscle; (2) humeral head tendon of the flexor carpi ulnaris muscle; (3) accessory carpal bone; and (4) peritendinous anechoic fluid and adjacent hypoechoic soft‐tissue thickening.

Lesions were localized to areas 13 and 14, corresponding to the FCUM insertion. All five exhibited musculotendinous thickening with heterogeneous echotexture and hypoechoic to mixed echogenicity. Head‐specific differentiation was possible in sagittal and transverse views and was most consistently assessed on sagittal views: both the humeral and ulnar heads were affected in two carpi, only the humeral head in two, and only the ulnar head in one carpus (Figure 8).

#### 3.7.6 Other Structures

Less frequently affected anatomic structures included the extensor digitorum communis muscle (*n* = 1/48; 2%), extensor carpi radialis muscle (*n* = 3/48; 6%), superficial digital flexor muscle (*n* = 2/48; 4%), deep digital flexor muscle (*n* = 2/48; 4%), and the medial collateral ligament (*n* = 1/48; 2%).

Radiographic and ultrasonographic findings varied depending on the anatomic structure involved but were consistently compatible with musculotendinous or ligamentous lesions (Supporting Information S2–S4).

### 3.8 Soft Tissue Volume Changes

Soft tissue swelling was identified in 46 of 48 (96%) carpi by either radiography or ultrasound. Radiographic assessment allowed only a binary distinction between normal and increased soft tissue volume, without further characterization of subcutaneous or deeper structures. In contrast, ultrasound allowed detailed differentiation among subcutaneous thickening, musculotendinous abnormalities, and joint effusion. The distribution pattern varied by diagnostic category and corresponded to the affected anatomic structure and its underlying pathology (Supporting Information S5–S8). Subcutaneous thickening with heterogeneous echogenicity was present in 27 of 48 (56%) carpi and was most commonly associated with musculotendinous lesions.

On ultrasound, subcutaneous thickening exhibited two recurring morphologic appearances: (i) a reticular, “honeycomb‐like” pattern characterized by fine hyperechoic septa surrounding small hypoechoic lobules, and (ii) a more uniform, diffuse increase in echogenicity with partial or complete loss of the normal lobulated subcutaneous fat architecture. Both appearances were nonshadowing and showed absent or minimal color or power Doppler flow.

Both morphologic appearances were observed, and in several cases the features of the two patterns overlapped, precluding clear differentiation between them on ultrasound. Accordingly, no etiologic assignment (e.g., interstitial edema vs. subcutaneous fibrosis) was made in Results section.

These subcutaneous alterations were observed across all diagnostic categories and were therefore analyzed collectively.

## 4 Discussion

This study represents the first comprehensive ultrasonographic case series across the spectrum of canine carpal disorders, with final case classification based on a composite reference standard and clinical follow‐up. By systematically characterizing ultrasonographic features across a broad range of pathologies—including musculotendinous disorders, inflammatory arthropathies, neoplasms, and bone lesions—it provides reference data for clinical use. Previous studies have focused on normal ultrasonographic anatomy [50, 51, 52, 54], experimental joint effusion [53], or isolated entities such as stenosing tenosynovitis of the abductor pollicis longus [55] and flexor carpi ulnaris tendinopathy [21, 22]; however, a structured ultrasound case series across the confirmed spectrum of canine carpal disorders has not previously been published.

Ultrasonography provided compartmental and structure‐specific information for the evaluation of carpal swelling. Radiography documents swelling but cannot localize it [39], whereas ultrasound localized abnormalities to subcutaneous, musculotendinous, periarticular, or intra‐articular compartments, thereby narrowing the differential diagnoses. Inflammatory arthropathies more often presented with generalized swelling, whereas the other diagnostic categories typically localized to the primarily affected structure [4, 12, 13, 32, 35, 55, 56, 57].

Subcutaneous changes were frequent concurrent findings and most commonly appeared as a reticular “honeycomb‐like” pattern and/or as a diffuse increase in echogenicity, often overlapping within the same region. Given the minimal thickness of the subcutaneous tissues in the carpal region and the overlap in sonographic appearance, reliable differentiation among edema, fibrosis, and cellulitis was not possible based on ultrasonographic appearance alone. Accordingly, no etiologic assignment was made in the Results section. In clinical interpretation, however, these nonspecific subcutaneous changes were considered most compatible with edema unless infection was confirmed by additional diagnostic testing, in which case the findings were classified as cellulitis. Color Doppler hyperemia was inconsistent, likely reflecting technical limitations and/or the low vascularity typically present in noninfectious edema [58, 59]. Future studies using ultrahigh‐frequency transducers [60], B‐flow, or contrast‐enhanced ultrasound may improve subcutaneous characterization [59, 61].

Musculotendinous disorders were the predominant category, with stenosing tenosynovitis of the abductor pollicis longus most frequent. Clinical, radiographic, and ultrasonographic findings were consistent with previous descriptions [23, 55, 62, 63]. Compared with radiography, ultrasound provided more detailed assessment, particularly for tendon‐sheath thickening, fiber irregularity, and peritendinous changes [55]. In contrast to earlier reports suggesting an association with agility or working dogs [55], this could not be confirmed in our cohort. This difference may reflect regional population and referral patterns.

Desmopathy of the accessoriometacarpal ligaments IV and V represented another frequent carpal disorder and is discussed here together because of their close anatomic relationship and frequent co‐involvement in the hyperextension injury complex [4, 33, 34, 64]. Many dogs with suspected hyperextension injury were managed during the emergency phase and were therefore excluded from the present cohort. In the enrolled cases, ultrasound demonstrated desmopathy and rupture of the accessoriometacarpal ligaments IV and V, despite its limited ability to fully characterize associated fractures; to the authors’ knowledge, this study provides the first ultrasonographic description of altered accessoriometacarpal ligaments IV and V in dogs (Figure 7). Focused prospective studies on the hyperextension injury complex are warranted.

Joint effusion, together with arthrocentesis and synovial fluid analysis, is central to the diagnosis and classification of joint disease [65], yet it may be difficult to detect by palpation, and its absence can delay arthrocentesis [30]. Previous work on carpal effusion in dogs was limited to experimental injection models [53]; to the authors’ knowledge, this study provides the first ultrasonographic description of naturally occurring carpal effusion in clinical patients (Figure 2). A consistent sign was dorsal displacement of the antebrachiocarpal fat pad; excessive transducer pressure should be avoided because it can redistribute fluid and reduce sensitivity. In clinically equivocal cases, dorsal as well as palmarolateral scans may be useful [53]. With the high‐frequency transducer used here, ultrasound can assess effusion and superficial capsular and synovial changes; capsular distension/capsulitis may contribute to pain given the dense sensory innervation of the fibrous capsule relative to the synovial lining, while synovitis may also be pain‐associated [66, 67]. In the present study, pain was recorded clinically (not during ultrasonography), and beyond effusion‐related capsular distension, no overt capsular thickening or synovial proliferative changes were identified. Future studies should standardize documentation and grading of carpal effusion using ultrahigh‐frequency transducers [60].

Ultrasonography was useful for detecting and anatomically localizing focal soft‐tissue masses. All six lesions were identified as mass lesions and confirmed as neoplasms cytologically or histopathologically. Sonographic features included heterogeneous echotexture, mixed echogenicity, absence of acoustic shadowing, poor‐to‐moderate margin definition, and commonly increased vascularity; however, these features can overlap with nonneoplastic conditions, including granulomatous disease [37]. Importantly, ultrasonography did not allow reliable benign–malignant classification; therefore, definitive classification requires tissue sampling. Given the rarity of canine carpal tumors [24, 25, 27, 28], contrast‐enhanced ultrasonography may clarify vascularity and infiltration patterns in future studies.

Formal diagnostic accuracy metrics (e.g., sensitivity and specificity) could not be calculated because of the observational design, heterogeneous case mix, and absence of a standardized control group; ultrasonography and cross‐sectional imaging were obtained only when clinically indicated, and contralateral comparison was not consistently available. Because validated quantitative cutoffs for the canine carpus are lacking, findings were graded using a predefined qualitative 0–3 scale rather than fixed numeric thresholds, with contralateral assessment applied qualitatively when available. Despite these limitations, the present data support a substantial practical diagnostic role for ultrasonography in the evaluation of canine carpal disease.

The integrated cross‐sectional imaging findings indicate that cross‐sectional imaging primarily refined lesion extent and osseous detail in selected cases, particularly in bone lesions including fractures, but did not consistently change the primary diagnosis established by combined radiography and ultrasonography.

## 5 Conclusion

Radiography and high‐frequency ultrasonography should be used in combination as a practical first‐line imaging approach for canine carpal disease. Ultrasonography is particularly useful for detecting carpal effusion and may facilitate timely arthrocentesis when clinical assessment is equivocal. CT or MRI should be reserved for selected cases, particularly those with complex, equivocal, or predominantly osseous disease.

## Author Contributions

*Conception and design*: **Sven Klußmann** and **Andreas Brühschwein**. *Acquisition of data*: Sven Klußmann, Andreas Brühschwein, and **Andrea Meyer‐Lindenberg**. *Analysis and interpretation of data*: Sven Klußmann, Andreas Brühschwein, and Andrea Meyer‐Lindenberg. *Drafting the article*: Sven Klußmann. *Reviewing the article for intellectual content*: Sven Klußmann and Andreas Brühschwein. *Final approval of the completed article*: Sven Klußmann, Andrea Meyer‐Lindenberg, and Andreas Brühschwein. *Agreement to be accountable for all aspects of the work in ensuring that questions related to the accuracy or integrity of any part of the work are appropriately investigated and resolved*: Sven Klußmann, Andreas Brühschwein, and Andrea Meyer‐Lindenberg.

## Conflicts of Interest

The authors declare no conflicts of interest.

## Disclosure

These results have not been previously presented or published. No reporting checklist was used.

## Data Accessibility Statement

The data that support the findings of this study are available from the corresponding author, S.K.

## Supporting information




**Supporting File**: vru70239‐sup‐0001‐SuppMat.docx.
