# Supplementary material for: Ultrasonography in Canine Carpal Disorders
Source: Vet Radiol Ultrasound. 2026 Sep 17;67(5):e70239. doi: 10.1111/vru.70239 (PMC13586417; doi:10.1111/vru.70239)
Supplement: Supplementary file 1 [file VRU-67-0-s001.docx]

SUPPLEMENT 1

Summary of the clinical parameters per category and carpus.

ABCJ: Antebrachiocarpal joint; MCJ: Mediocarpal joint; CMCJ: Carpometacarpal joint;

IMPA: Immune-mediated polyarthritis.

SUPPLEMENT 2

Radiographic imaging findings per category and carpus.

ABCJ: Antebrachiocarpal joint; MCJ: Mediocarpal joint; CMCJ: Carpometacarpal joint. AMLIV: Accessoriometacarpal ligament IV; AMLV: Accessoriometacarpal ligament V; APL: Abductor pollicis longus; FCR: Flexor carpi radialis; FCU: Flexor carpi ulnaris; LML: Lateral collateral ligament; MCL: Medial collateral ligament.

SUPPLEMENT 3

Ultrasonographic imaging findings per category and carpus.

ABCJ: Antebrachiocarpal joint; MCJ: Mediocarpal joint; CMCJ: Carpometacarpal joint.

SUPPLEMENT 4

Detailed ultrasonographic imaging findings per disordered anatomical structure (category musculotendinous disorders). AMLIV: Accessoriometacarpal ligament IV; AMLV: Accessoriometacarpal ligament V; APL: Abductor pollicis longus; DDFM: Deep digital flexor muscle; EDC: Extensor digitorum communis; ECR: Extensor carpi radialis; FCU: Flexor carpi ulnaris; MCL: Medial collateral ligament; SDFM: Superficial digital flexor muscle. TS: Tendon sheath.

SUPPLEMENT 5

Distribution of radiographically altered soft tissue volume per area 1-8 and category in %.

SUPPLEMENT 6

Distribution of ultrasonographically altered soft tissue volume per area 1-16 and category in %.

SUPPLEMENT 7

Distribution of radiographically altered soft tissue volume per anatomical structure and area 1-8 in %. AMLIV: Accessoriometacarpal ligament IV; AMLV: Accessoriometacarpal ligament V; APLM: Abductor pollicis longus muscle; ECRM: Extensor carpi radialis muscle; EDCM: Extensor digitorum communis muscle; FCUM: Flexor carpi ulnaris muscle; SDFM: Superficial digital flexor muscle; DDFM: Deep digital flexor muscle; MCL: Medial collateral ligament.

SUPPLEMENT 8

Distribution of ultrasonographically altered soft tissue volume per anatomical structure and area 1-16 in %. AMLIV: Accessoriometacarpal ligament IV; AMLV: Accessoriometacarpal ligament V; APLM: Abductor pollicis longus muscle; ECRM: Extensor carpi radialis muscle; EDCM: Extensor digitorum communis muscle; FCUM: Flexor carpi ulnaris muscle; SDFM: Superficial digital flexor muscle; DDFM: Deep digital flexor muscle; MCL: Medial collateral ligament.
